# Use of a Remote Oncology Pharmacy Service Platform for Patients With Cancer During the COVID-19 Pandemic: Implementation and User Acceptance Evaluation

**DOI:** 10.2196/24619

**Published:** 2021-01-21

**Authors:** Zhuo-Jia Chen, Wei-Ting Liang, Qing Liu, Rong He, Qian-Chao Chen, Qiu-Feng Li, Yao Zhang, Xiao-Dong Du, Ying Pan, Shu Liu, Xiao-Yan Li, Xue Wei, He Huang, Hong-Bing Huang, Tao Liu

**Affiliations:** 1 Department of Pharmacy Sun Yat-sen University Cancer Center Guangzhou China; 2 Department of Information Sun Yat-sen University Cancer Center Guangzhou China; 3 Department of Medicine Sun Yat-sen University Cancer Center Guangzhou China

**Keywords:** COVID-19, cancer patients, remote pharmacy, service platform, implementation, oncology, pharmacy, online platform, cancer, health management, app, online hospital, acceptance, impact

## Abstract

**Background:**

The COVID-19 outbreak has increased challenges associated with health management, especially cancer management. In an effort to provide continuous pharmaceutical care to cancer patients, Sun Yat-sen University Cancer Center (SYSUCC) implemented a remote pharmacy service platform based on its already existing web-based hospital app known as Cloud SYSUCC.

**Objective:**

The aim of this study was to investigate the characteristics, acceptance, and initial impact of the Cloud SYSUCC app during a COVID-19 outbreak in a tertiary cancer hospital in China.

**Methods:**

The total number of online prescriptions and detailed information on the service were obtained during the first 6 months after the remote service platform was successfully set up. The patients’ gender, age, residence, primary diagnosis, drug classification, weekly number of prescriptions, and prescribed drugs were analyzed. In addition, a follow-up telephonic survey was conducted to evaluate patients’ satisfaction in using the remote prescription service.

**Results:**

A total of 1718 prescriptions, including 2022 drugs for 1212 patients, were delivered to 24 provinces and municipalities directly under the Central Government of China between February 12, 2020, and August 11, 2020. The majority of patients were female (841/1212, 69.39%), and 90.18% (1093/1212) of them were aged 31-70 years old. The top 3 primary diagnoses for which remote medical prescriptions were made included breast cancer (599/1212, 49.42%), liver cancer (249/1212, 20.54%), and thyroid cancer (125/1212, 10.31%). Of the 1718 prescriptions delivered, 1435 (83.5%) were sent to Guangdong Province and 283 (16.5%) were sent to other provinces in China. Of the 2022 drugs delivered, 1012 (50.05%) were hormonal drugs. The general trend in the use of the remote prescription service declined since the 10th week. A follow-up telephonic survey found that 88% (88/100) of the patients were very satisfied, and 12% (12/100) of the patients were somewhat satisfied with the remote pharmacy service platform.

**Conclusions:**

The remote pharmacy platform Cloud SYSUCC is efficient and convenient for providing continuous pharmaceutical care to patients with cancer during the COVID-19 crisis. The widespread use of this platform can help to reduce person-to-person transmission as well as infection risk for these patients. Further efforts are needed to improve the quality and acceptance of the Cloud SYSUCC platform, as well as to regulate and standardize the management of this novel service.

## Introduction

China and the rest of the world are experiencing an outbreak of the highly infectious, novel COVID-19 [1-3]. To effectively combat the COVID-19 pandemic, Chinese health authorities have effected a series of preventive and control measures since January 21, 2020, including restrictions on people’s movement, reduced transportation, entry and exit controls for towns and villages, and isolation requirements for travelers between various parts of the country [4]. However, for patients with cancer, restrictions on movement could result in disease upstaging and have considerable impact on cancer-specific death rates due to delayed diagnoses and the lack of or suboptimal cancer management. Therefore, the management of patients with cancer has become a crucial issue in cities with large-scale outbreaks of COVID-19 [5-7].

Since May 24, 2018, Sun Yat-sen University Cancer Center (SYSUCC) in China has implemented a hospital-based, mobile app called Cloud SYSUCC that enables remote medication consultation between doctors and patients, available both on Android and iOS mobile operating systems. This app includes 2 introductory screens: one for the medical staff (ie, therapeutic interface) and the other for patients (ie, patient interface; see Figure S1 in Multimedia Appendix 1). The therapeutic interface has been designed for doctors to check a patient’s medical history, communicate with them, and order examinations; however, it did not provide access to medication prescriptions. For this reason, a multidisciplinary working group comprising senior hospital pharmacists, clinical experts, and information technology engineers was formed on January 23, 2020, to set up a remote pharmacy service platform based on the Cloud SYSUCC app. This platform would enable remote services such as web-based consultation, prescription, dispensation, and home delivery of oral anticancer medications, as well as offer standard administration instructions for the use of these drugs. In this paper, we introduce this remote pharmacy service system and describe its development and implementation in a tertiary cancer hospital in China.

To explore the advantages of the remote pharmacy service system for patients with cancer during public health emergencies, we analyzed the online prescriptions of outpatients at SYSUCC in Guangzhou City, Guangdong Province, China. We collected data from the first 6 months after the remote service platform was successfully set up, and we analyzed the characteristics, user acceptance, and initial impact of this new bundled approach during the COVID-19 outbreak.

## Methods

### Platform Development

The remote pharmacy service platform provides services to the public via the Cloud SYSUCC app, which has been deployed on a public cloud. A private cloud connects the public cloud with the internal hospital information system using a web services internet tool.

### Data Sources

The total number of online prescriptions and their detailed information were obtained automatically from the hospital information system of Cloud SYSUCC. We collected data from February 12, 2020, to August 11, 2020. Patient’s gender, age, residence, primary diagnosis, drug classification, weekly number of prescriptions, and prescribed drugs were included in the analysis. Furthermore, we also conducted a telephonic survey to evaluate patients’ satisfaction with using the remote prescription service. We used SPSS software (version 16.0; IBM Corp) to summarize and analyze the data.

## Results

### Real-Time Medication Consultation

**Figure 1:**
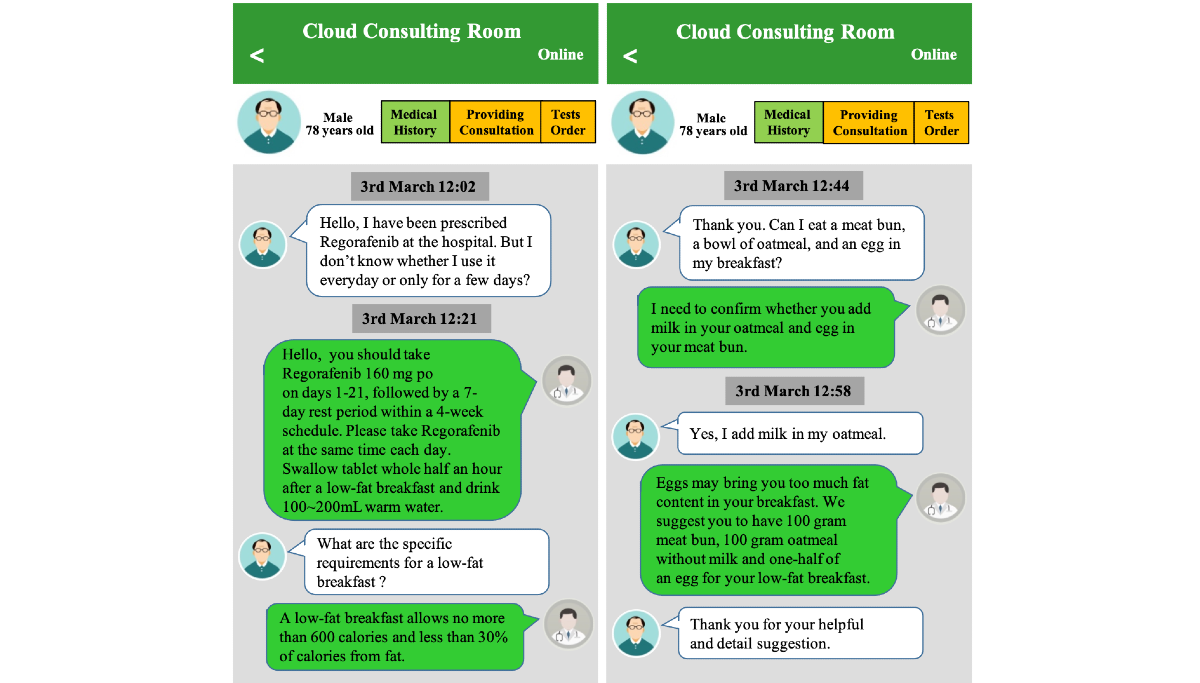
Screenshot of real-time medication consultation between clinical pharmacists and patients.

We integrated the therapeutic interface of Cloud SYSUCC with the internal hospital information system by using a web services internet tool. Each medical staff, including pharmacists, could access the platform by using their unique staff identification number and password. This integration allows clinical pharmacists to provide real-time patient consultation and check the patient’s medical history when necessary (Figure 1). They can also query the patient’s consultations status (eg, pending or reviewed), and their observations can be documented in the electronic clinical history.

### Online Prescriptions

Considering patients’ safety, effectiveness, and emergency medical needs, the Pharmacy and Therapeutics Committee at SYSUCC has approved 22 oral anticancer drugs and 9 adjuvant medicines; these medications are currently listed in the Cloud SYSUCC app (Table 1). Doctors can prescribe the required drugs through the app in compliance with the following guidelines. First, for prescription of chemotherapeutic drugs, their potential adverse events should be considered and adequate surveillance should be allowed; the duration of these prescriptions should not exceed 14 days. Second, nonchemotherapeutic drugs can be prescribed for a duration not exceeding 30 days. Third, to address the need to balance the safety and feasibility of chemotherapeutic drugs, each patient is allowed 2 remote chemotherapy prescription cycles, after which they are required to visit the hospital for adequate examinations before they can continue using the Cloud SYSUCC app. All prescriptions are pharmaceutically verified by an automated drug rationality review system integrated into our cloud-based hospital information system.

**Table 1:** Anticancer drugs (n=22) and adjuvant medicines (n=9) listed in the Cloud SYSUCC app.

Drug type and name		Dosage form	Dosage strength	Drug quantity		Disease name		
**Hormonal** **drugs**								
	Exemestane	Tablet	25 mg	14		Breast cancer		
	Anastrozole	Tablet	1 mg	14		Breast cancer		
	Letrozole	Tablet	2.5 mg	30		Breast cancer		
	Toremifene	Tablet	60 mg	30		Breast cancer		
	Tamoxifen	Tablet	10 mg	60		Breast cancer		
	Medroxyprogesterone	Tablet	500 mg	30		Breast cancer		
	Megestrol acetate	Tablet	80 mg	24		Breast cancer, endometrial cancer		
	Bicalutamide	Tablet	50 mg	28		Prostate cancer		
	Levothyroxine sodium	Tablet	50 µg	100		Thyroid cancer		
**Calcium preparations**								
	Calcium carbonate	Tablet	1.5 g	30		Osteoporosis		
	Alfacalcidol	Capsule	1 µg	10				
	Calcitriol	Capsule	0.25 µg	10				
	Calcium supplement with vitamin D	Tablet	750 mg	60				
**Antiviral drugs**								
	Entecavir	Tablet	0.5 mg	7		Type B viral hepatitis		
	Tenofovir	Tablet	0.3 g	30				
	Telbivudine	Tablet	600 mg	7				
	Lamivudine	Tablet	0.1 g	14				
	Adefovir dipivoxil	Tablet	10 mg	14				
**Alkylating drugs**								
	Cyclophosphamide	Tablet	50 mg	24		Hodgkin disease, acute myeloid leukemia, breast cancer		
	Temozolomide	Capsule	20 mg	5		Glioblastoma multiforme		
	Temozolomide	Capsule	100 mg	5		Anaplastic astrocytoma		
**Antimetabolite drugs**								
	Capecitabine	Tablet	500 mg	12		Colorectal cancer		
	Tegafur	Tablet	50 mg	100		Gastric cancer, colorectal cancer, breast cancer		
	Tegafur/Uracil Monopotassium	Tablet	162 mg	20		Gastric cancer, colorectal cancer, breast cancer		
	Tegafur/Gimeracil/Oteracil Monopotassium	Capsule	20 mg	140		Gastric cancer		
**Angiogenesis inhibitors**								
	Thalidomide	Capsule	25 mg	48		Multiple myeloma		
**Kinase inhibitors**								
	Gefitinib	Tablet	250 mg	10		Lung cancer		
	Imatinib	Tablet	100 mg	60		Gastrointestinal stromal tumor, lymphoblastic leukemia		
	Icotinib	Tablet	125 mg	21		Lung cancer		
	Erlotinib	Tablet	100 mg	30				
			150 mg	7				
	Apatinib	Tablet	250 mg	10		Gastric adenocarcinoma, gastroesophageal conjunctive adenocarcinoma		
	Afatinib	Tablet	30 mg	7		Lung cancer		
			40 mg	7				

### Drug Dispensation and Home Delivery Service

**Figure 2:**
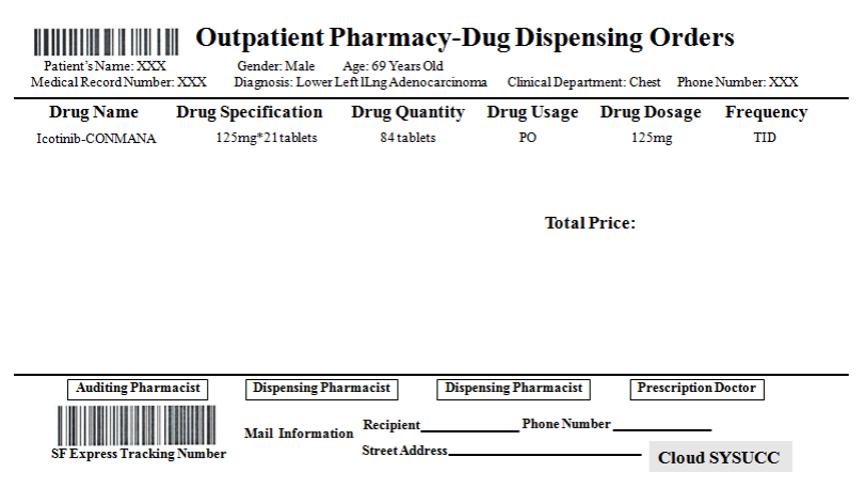
Example of a drug dispensing order generated using the Cloud SYSUCC app.

Once an online prescription is generated from the Cloud SYSUCC app, an alert system linked to the SYSUCC drug dispensing system is initiated. Then, the frontline pharmacists can print the drug dispensing orders with an automatically generated delivery service tracking number (SF Express Group Co., Ltd.; Figure 2). All drug orders are double-checked before being transferred to SF Express, which provides a quick, cost-effective, and meticulous Mainland China Express Service to ensure safe and efficient delivery. Patients can view live updates by using the tracking information of the drug orders they have placed via the Cloud SYSUCC app. To ensure the drugs are delivered correctly to patients, SF Express delivery agents verify the identity of the patients before handing out the packages. If the delivery of packages takes more than 3 days, the patients can simply request a refund for the express order charges.

### Standard Administration Instructions

**Figure 3:**
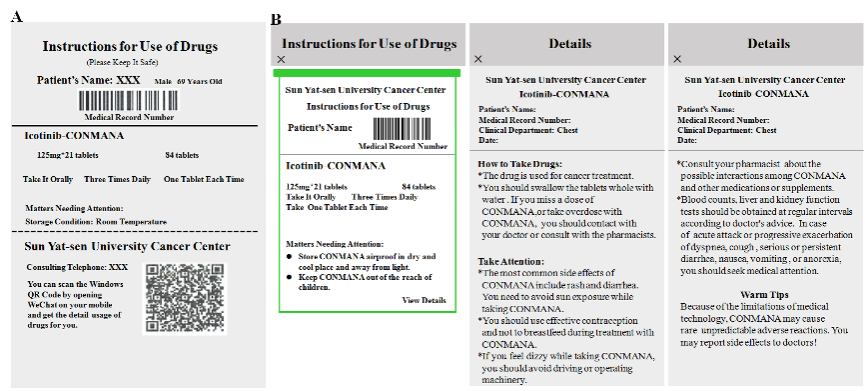
Screenshots of drug instructions shared with patients ordering drugs via the Cloud SYSUCC app: (A) printed version and (B) electronic version.

To ensure safe handling of oral anticancer drugs by patients at home, clinical pharmacists have undertaken the following essential measures: (1) prescription directions are printed on each drug box; (2) detailed directions for the usage of drugs are made available on the patient’s interface once the drugs are ordered (Figure 3A); and (3) patients can use their mobile phone’s camera to scan a QR (quick response) code that will prompt a WeChat (Tencent Tech Shenzhen Co., Ltd.) window on their phone, wherein also detailed drug usage directions will be provided. These measures will ensure patients have easy access to the detailed instructions whenever they need (Figure 3B). If patients have any concerns regarding the drugs, they can consult with their treating doctors or clinical pharmacists using the Cloud SYSUCC app.

### Patient Demographics and Distribution of Online Prescriptions

**Figure 4:**
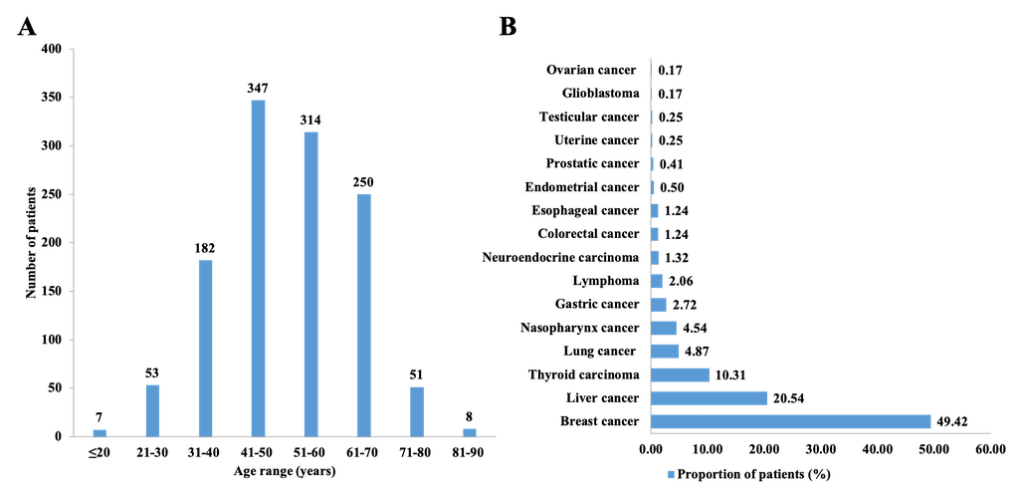
Distribution of patients by age (A) and primary diagnosis (B) using data from the Cloud SYSUCC app (N=1212).

During the study period (February 12 to August 11, 2020), a total of 1718 prescriptions, including 2022 drugs for 1212 patients, were delivered to 24 provinces and municipalities directly under the Central Government of China. The patient demographics are shown in Table 2. The majority of patients were female (841/1212, 69.39%). The mean age of patients was 51.26 (SD 12.29) years (age range: 12-87), and 90.18% (1093/1212) of the patients were aged between 31 and 70 years old (Figure 4A). The distribution of patients based on their primary diagnosis showed that breast cancer, liver cancer, and thyroid cancer were the 3 most common types of cancers treated (49.42%, 20.54%, and 10.31%, respectively; Figure 4 B) with prescriptions using this remote platform.

**Table 2:** Characteristics of patients who received online prescriptions from the Cloud SYSUCC app (N=1212).

Characteristic		Value
**Gender, n (%)**		
	Male	371 (30.61)
	Female	841 (69.39)
Age (years), median (range)		51 (12-87)
**Age group (years), n (%)**		
	0-10	0 (0.00)
	10-20	7 (0.58)
	21-30	53 (4.37)
	31-40	182 (15.02)
	41-50	347 (28.63)
	51-60	314 (25.91)
	61-70	250 (20.63)
	71-80	51 (4.21)
	81-90	8 (0.66)
**Primary diagnosis, n (%)**		
	Breast cancer	599 (49.42)
	Liver cancer	249 (20.54)
	Thyroid carcinoma	125 (10.31)
	Lung cancer	59 (4.87)
	Nasopharyngeal cancer	55 (4.54)
	Gastric cancer	33 (2.72)
	Lymphoma	25 (2.06)
	Neuroendocrine carcinoma	16 (1.32)
	Colorectal cancer	15 (1.24)
	Esophageal cancer	15 (1.24)
	Endometrial cancer	6 (0.50)
	Prostatic cancer	5 (0.41)
	Uterine cancer	3 (0.25)
	Testicular cancer	3 (0.25)
	Glioblastoma	2 (0.17)
	Ovarian cancer	2 (0.17)

**Figure 5:**
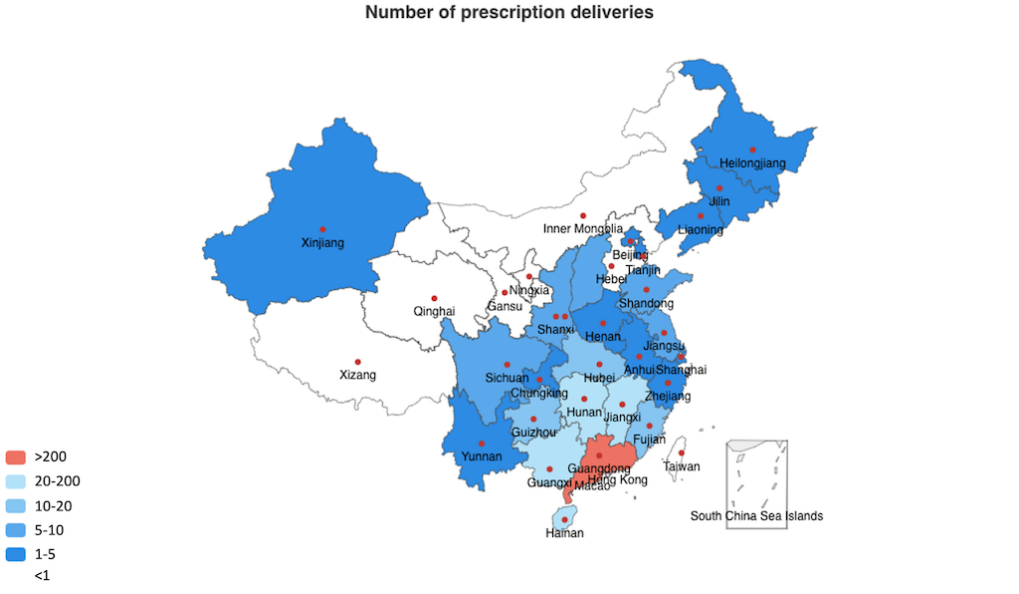
Regional distribution of prescription deliveries via the Cloud SYSUCC app (N=1718).

Of the 1718 delivered prescriptions, 1435 (83.5%) prescriptions were delivered to Guangdong Province, including 437 (25.4%) in Guangzhou city, and the remaining 283 (16.5%) prescriptions were delivered to other provinces in China (Figure 5). The top 5 provinces for out-of-province prescription deliveries were Hunan, Guangxi, Jiangxi, Hainan, and Fujian, all of which are located in southern China (Figure 5).

**Figure 6:**
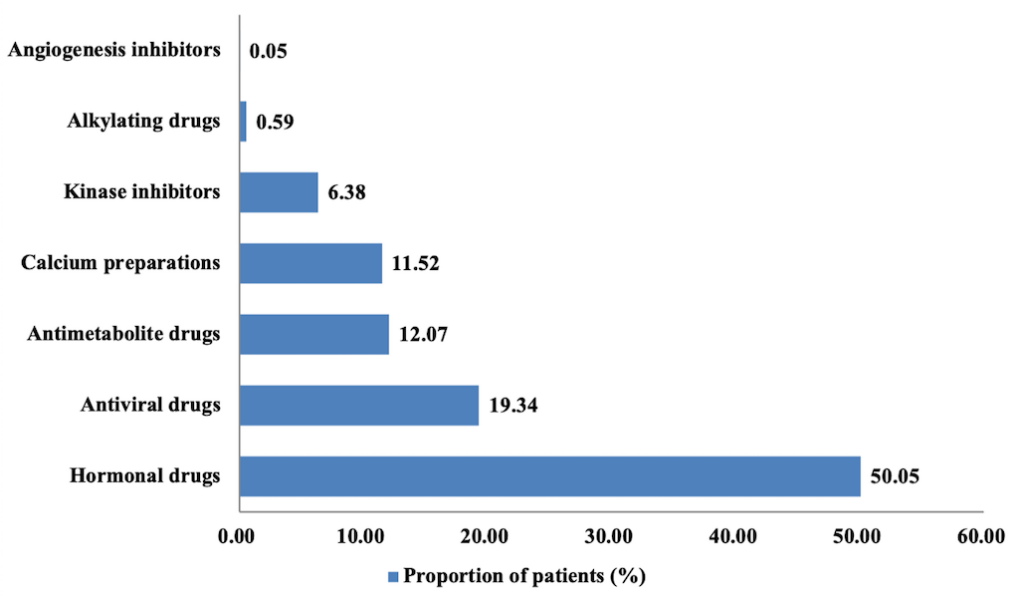
Distribution of online prescriptions by classification of drugs ordered via the Cloud SYSUCC app (N=2022).

Figure 6 shows the distribution of drugs delivered (mean 1.19, SD 0.46; range: 1-6) to the 1212 patients based on drug classification. Among the 2022 drugs delivered, 1012 (50.05%) were hormonal drugs. Most hormonal drugs were used to treat breast cancer and thyroid cancer, which was consistent with the distribution of the patients by primary diagnosis.

**Figure 7:**
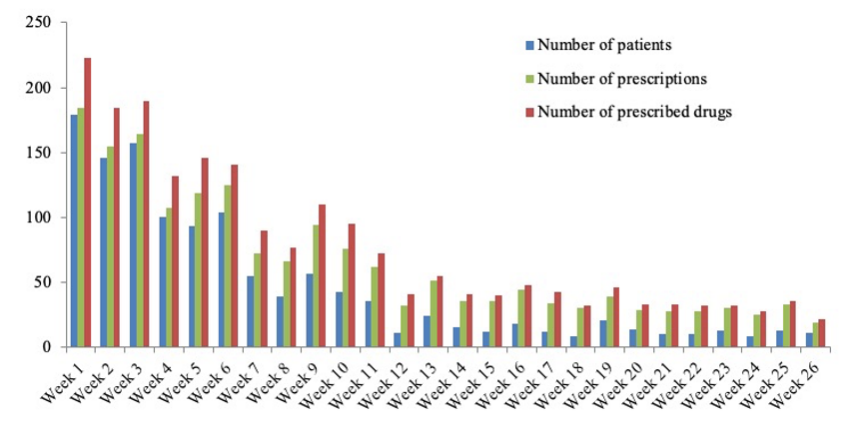
Weekly number of patients who used the remote prescription system, medication prescriptions ordered, and prescribed drugs delivered via the Cloud SYSUCC app since February 12, 2020.

The weekly number of prescriptions, prescribed drugs, and patients pertaining to these prescriptions are summarized in Figure 7. As the COVID-19 outbreak was brought under control in China, the general trend in the use of the remote prescription service considerably declined. The total number of patients who used the remote prescription service in the first 9 weeks of the outbreak was 930; this number was 3 times higher than the number of patients who used the service in the following 17 weeks.

### Telephonic Patient Satisfaction Survey Evaluating the Remote Prescription Service

A small-scale telephonic survey was conducted to obtain patients’ views about the remote prescription service. A total of 100 patients were randomly selected from the total sample of 1212 patients over a 6-month period. These patients were contacted 7 days after their prescriptions were delivered. This time lag between prescription delivery and inquiry was intended to allow patients sufficient time to receive the drugs and reflect on their experiences. The survey contained only 1 simple question: “What do you think about the remote prescription service?” Patients were asked to choose from the following 5 multiple choice answers: (1) satisfied, (2) somewhat satisfied, (3) neutral, (4) somewhat dissatisfied, and (5) very dissatisfied. The survey question was very specific to the remote prescription service so that patients were clear about what was being asked and could accurately express their actual experience. If a selected patient declined to participate in the survey, another patient would be chosen to substitute him or her. The use of randomization to select the survey sample was to ensure a general representation of the population of study patients using the remote prescription service. Thus, bias in the patient sample, such as age, gender, and social class was reduced. We believe that this randomization of the sample enhanced the external validity of the survey results. The telephonic survey showed that 88% (88/100) of the participants were satisfied, whereas 12% (12/100) of them were somewhat satisfied. None of the patients selected a neutral, somewhat dissatisfied, or very dissatisfied response. Overall, these findings suggest a high level of user satisfaction with our remote pharmacy service platform.

## Discussion

### Principal Findings

We conducted a pilot evaluation of the remote pharmacy service platform in a tertiary cancer hospital in Guangzhou, China, during the first 6 months after work resumption. By September 20, 2020, the rapid spread of COVID-19 had resulted in 30,675,675 cases and 954,417 deaths worldwide. Traditional hospitals required patients to visit the hospitals for obtaining medications, which could potentially cause more infections and lead to severe health deterioration during an ongoing epidemic, particularly for patients with cancer who have a suppressed immune system. Studies have shown that patients with cancer have a higher risk of severe events than do other patients [8,9]. Moreover, a delayed diagnosis and suboptimal cancer management due to the pandemic could be life-threatening for these patients [5,6]. Therefore, access to timely medications has become a crucial issue during large-scale outbreaks of COVID-19. Our remote pharmacy service platform can prove beneficial for such use, with a good satisfactory response from patients with cancer. To our knowledge, this remote pharmacy service platform for patients with cancer is the first such information system introduced worldwide, comprising a collaborative mechanism that integrates the Cloud SYSUCC app with the internal hospital information system and facilitates remote consultation service by using a standardized format for ethical drug prescription and standard administration instructions.

From February 12, 2020, to August 11, 2020, a total of 60,968 users used the Cloud SYSUCC app, an average of 500 users per day. This resulted in 1718 prescriptions and US $3054 million in drugs fees. Most of the medical consultations were for prehospital services such as cancer diagnosis, cancer type detection, and cancer treatment. The number of online prescriptions recorded increased from the first week investigated to the 6th week (Figure 7), which indicates the acceptance of Cloud SYSUCC app among the users. The decline in prescription numbers since the 7th week might be the result of 2 factors, namely, the revision in the Cloud SYSUCC registration fee from US $7 to US $70 per user and the fact that the COVID-19 outbreak was brought under control.

Most patients using the prescription service were female with a breast cancer diagnosis, aged between 31 and 70 years, and had an existing prescription for hormonal drugs. Since hormone-receptor-positive, early-stage breast cancer is a chronic disease, continuous daily dosing of hormone therapy after surgery is very important in these cases. However, at the same time, hormone therapy may cause serious adverse reactions, not only affecting the patient’s quality of life but also eventually leading to therapeutic resistance. Therefore, timely evaluation and adaptation of treatment is needed for these patients. Hence, the Pharmacy and Therapeutics Committee at SYSUCC has authorized doctors to prescribe the required drugs through the Cloud SYSUCC app provided that the prescriptions are compliant with specific guidelines to balance safety and feasibility of the use of anticancer drugs. Moreover, exclusive medical education campaigns are carried out for older patients who were recommended to stay at home and avoid contact with other people for an extended period during the COVID-19 outbreak [10]. Our results showed that only 4.87% of the patients requiring online prescriptions were aged ≥70 years (Table 2). This may be due to the differences in public acceptance and uncertainty of physical conditions for older-aged populations.

### Future Prospects

Thus far, the COVID-19 outbreak in China has been under control, and there is no rapid increase in the number of new cases. However, the possibility of a renewed spike in COVID-19 cases in individual regions cannot be ruled out. Therefore, more effort is needed to make better use of the Cloud SYSUCC–based remote pharmacy service during and after the current pandemic. Simple and clear instructions are necessary to improve acceptance of this platform by older patients. Improving financial support, such as by reducing the Cloud SYSUCC registration fee, can also promote increased adoption of the platform by the public. Moreover, timely web-based pharmaceutical care interventions and patient monitoring are required to ensure the safety of patients with cancer.

### Conclusions

Currently, the world is fighting against the COVID-19 pandemic, which has affected over 200 countries and regions. The findings of this study suggest that the Cloud SYSUCC remote pharmacy platform is efficient and convenient for enabling continuous pharmaceutical care to patients with cancer. The widespread use of this platform can help reduce both person-to-person transmission of COVID-19 and the infection risk for patients with cancer.

## Appendix

Multimedia Appendix 1 Screenshot of the home page of the Cloud SYSUCC app: (A) therapeutic interface and (B) patient interface.

## Abbreviations

SYSUCC: Sun Yat-sen University Cancer Center
