# Supplementary material for: Use of a Remote Oncology Pharmacy Service Platform for Patients With Cancer During the COVID-19 Pandemic: Implementation and User Acceptance Evaluation
Source: J Med Internet Res. 2021 Jan 21;23(1):e24619. doi: 10.2196/24619 (PMC7894743; doi:10.2196/24619)
Supplement: Multimedia Appendix 1 [file jmir_v23i1e24619_app1.docx]

**
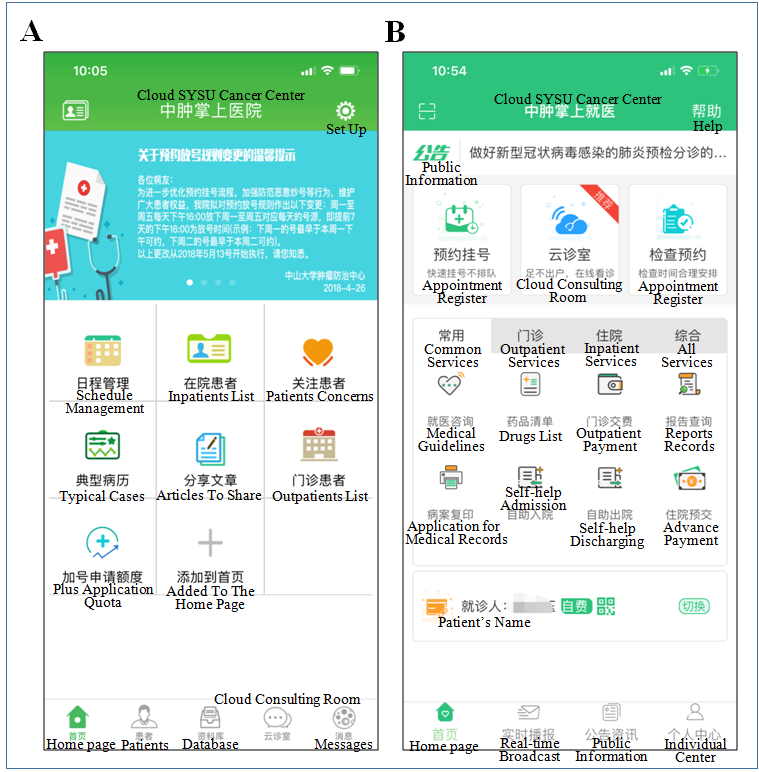
**

**Figure. S1 Screenshot of the Home Page of the Cloud SYSUCC app**

(A) the therapeutic interface; (B) the patient interface
